# Special Issue “Fiber Optic Sensors and Applications”: An Overview

**DOI:** 10.3390/s20123400

**Published:** 2020-06-16

**Authors:** Lei Wei, Swee Chuan Tjin

**Affiliations:** School of Electrical and Electronic Engineering and The Photonics Institute, Nanyang Technological University, 50 Nanyang Avenue, Singapore 639798, Singapore; esctjin@ntu.edu.sg

**Keywords:** fiber optic sensors, detection mechanisms, materials, applications

## Abstract

We present here the recent advance in exploring new detection mechanisms, materials, processes, and applications of fiber optic sensors.

## 1. Introduction

In this Special Issue, we aim to focus on all aspects of the recent research and development related to fiber optic sensors. Recent advances in fiber-based sensing technologies have enabled both fundamental studies and a wide spectrum of applications [1,2,3]. This Special Issue seeks to bring attention to the most recent results in the field of fiber optic sensors offered by their unique features and advantages, including new detection mechanisms, materials, processes, and applications.

## 2. Summary of Special Issue Papers

Below is a brief summary of all the articles covered in this Issue. 

In “High-Sensitivity, Large Dynamic Range Refractive Index Measurement Using an Optical Microfiber Coupler” [4], a sensing strategy was proposed by utilizing the unique property of the dispersion turning point in an optical microfiber coupler mode interferometer. As a result, high sensitivity of larger than 5327.3 nm/RIU was achieved in the whole refractive index range of 1.333–1.4186. This sensor offered good performance in narrow refractive index ranges with high resolution and high linearity. 

In “An Intra-Oral Optical Sensor for the Real-Time Identification and Assessment of Wine Intake” [5], an intra-oral optical fiber sensor was developed around an optical coupler topology and exemplified on the detection and assessment of wine intake. Its implementation exploited the advantages of fiber-optics sensing, and facilitated the integration into a mouthguard, holding considerable potential for real-time biomedical applications for the evaluation of risk factors in diet-related diseases. 

In “Laser-Induced Deposition of Carbon Nanotubes in Fiber Optic Tips of MMI Devices” [6], a laser-induced technique was developed to obtain the deposition of carbon nanotubes (CNTs) onto the fiber optics tips of multimode interference (MMI) devices. The laser-induced deposition of CNTs performed in water-based solutions generated nonuniform deposits, while the laser-induced deposition performed with methanol solutions generated uniform deposits over the fiber tip, indicating the crucial role of the solvent on the spatial features of the laser-induced deposition process. 

In “Fiber Link Health Detection and Self-Healing Algorithm for Two-Ring-Based RoF Transport Systems” [7], a two-ring-based radio over fiber (RoF) transport system with a two-step fiber link failure detection and self-healing algorithm was proposed to ensure quality of service (QoS) by automatically monitoring the health of each fiber link in the transport system and by resourcefully detecting, locating, and bypassing the blocked fiber links. Moreover, the proposed algorithm was able to find the blocked fiber links in the RoF transport system and animatedly adjust the status of preinstalled optical switches to restore all blocked network connections. 

In “Design and Implementation of a Novel Measuring Scheme for Fiber Interferometer Based Sensors” [8], a measuring scheme for fiber interferometer based sensors was proposed to detect the equivalent changes of optical power corresponding to the variation in measuring parameters, and a signal processing system was used to analyze the optical power changes and to determine the spectrum shifts. A sensing device on polymer microcavity fiber interferometer was taken as an example for constructing a measuring system capable of long-distance monitoring of the temperature and relative humidity. 

In “Nonlinearity Correction in OFDR System Using a Zero-Crossing Detection-Based Clock and Self-Reference” [9], a method for tuning nonlinearity correction in an optical frequency-domain reflectometry (OFDR) system was developed from the aspect of data acquisition and post-processing. The spatial resolution test and the distributed strain measurement test were both performed based on this nonlinearity correction method, which reduced the hardware and data burden for the system and has potential value for system integration and miniaturization. 

In “Dynamic Deformation Reconstruction of Variable Section WING with Fiber Bragg Grating Sensors” [10], a dynamic reconstruction algorithm based on the inverse finite element method and fuzzy network was proposed to sense the deformation of the variable-section beam structure. Considering the installation error of the fiber Bragg grating (FBG) sensor and the dynamic un-modeled error caused by the difference between the static model and dynamic model, the real-time measured strain was corrected using a solidified fuzzy network. 

In “Metal Forming Tool Monitoring Based on a 3D Measuring Endoscope Using CAD Assisted Registration” [11], a setup by combining a 3D measuring endoscope with a two-stage kinematic was demonstrated based on the projection of structured light, allowing time-effective measurements of larger areas. By the use of computer-aided design (CAD) data, registration was improved, allowing a detailed examination of local features like gear geometries while reducing the sensitivity to detect shape deviations.

In “A High Sensitivity Temperature Sensing Probe Based on Microfiber Fabry–Perot Interference” [12], a miniature Fabry–Perot temperature probe was designed by using polydimethylsiloxane to encapsulate a microfiber in one cut of hollow core fiber. The temperature sensing performance was experimentally demonstrated with a sensitivity of 11.86 nm/°C and an excellent linear fitting in the range of 43–50 °C, making it a promising candidate for exploring the temperature monitor or controller with ultrahigh sensitivity and precision.

In “Hybrid Plasmonic Fiber-Optic Sensors” [13], the development of plasmonics-based fiber-optic sensors was reviewed to reveal and explore the frontiers of such hybrid plasmonic fiber-optic platforms in various sensing applications. Coupled with the new advances in functional nanomaterials as well as fiber structure design and fabrication in recent years, new solutions continue to emerge to further improve the fiber-optic plasmonic sensors’ performances in terms of sensitivity, specificity, and biocompatibility. 

In “Carbon Allotrope-Based Optical Fibers for Environmental and Biological Sensing: A Review” [14], the development of carbon allotropes-based optical fiber sensors was reviewed. The first section provided an overview of four different types of carbon allotropes, including carbon nanotubes, carbon dots, graphene, and nanodiamonds. The second section discussed the synthesis approaches used to prepare these carbon allotropes, followed by some deposition techniques to functionalize the surface of the optical fiber, and the associated sensing mechanisms. Finally, a concluding section highlighting the technological deficiencies, challenges, and suggestions to overcome them was presented.

In “Relative Humidity Sensors Based on Microfiber Knot Resonators-A Review” [15], the recent research and development progress of relative humidity sensors using microfiber knot resonators (MKRs) were reviewed by considering the physical parameters of the MKR and coating materials sensitive to improve the relative humidity sensitivity. There are many advantages of the MKR, such as strong evanescent field, a high Q-factor, compact size, and high sensitivity provided a great diversity of sensing applications. The sensing performance of the MKR-based relative humidity sensors was also discussed, including sensitivity, resolution, and response time. 

In “Dual-Polarized Fiber Laser Sensor for Photoacoustic Microscopy” [16], the recent progress in fiber-laser-based ultrasound sensors for photoacoustic microscopy was reviewed, especially the dual-polarized fiber laser sensor with high sensitivity. The principle, characterization, and sensitivity optimization of this type of sensor were presented. In vivo experiments demonstrated its excellent performance in the detection of photoacoustic signals in optical resolution photoacoustic microscopy (OR-PAM). This review also summarized the representative applications of fiber laser sensors in OR-PAM and discussed their further improvements. 
